# Risk factors and methods of NSSI and suicide attempts among American Indian and Alaska native adolescents and young adults: A systematic review protocol

**DOI:** 10.1371/journal.pone.0358536

**Published:** 2026-09-15

**Authors:** Anvar Sadath, Yiwei Zhang, Praveen Kumar Rudra, Omer Farooq, Darian Draft, Susan Reay

**Affiliations:** 1 Grace Abbott School of Social Work, College of Public Affairs and Community Service, University of Nebraska at Omaha, Omaha, Nebraska, United States of America; 2 Department of Counseling, College of Education, Health, and Human Sciences, University of Nebraska at Omaha, Omaha, Nebraska, United States of America; 3 Criss Library, University of Nebraska at Omaha, Omaha, Nebraska, United States of America; Gachon University Gil Medical Center, KOREA, REPUBLIC OF

## Abstract

**Background:**

Suicide is a leading cause of premature mortality worldwide and a major public health concern among adolescents and young adults. In the United States, American Indian and Alaska Native (AI/AN) populations have the highest suicide rates of any racial or ethnic group, with suicide among the leading causes of death in AI/AN youth. Suicide attempts are a clinically significant outcome and a strong predictor of subsequent suicide death. To our knowledge, no prior systematic review has simultaneously focused on AI/AN adolescents and young adults while examining risk factors, suicide attempt methods, and indicators of clinical severity or lethality. Addressing this gap is critical for informing equitable suicide prevention strategies. This protocol describes a systematic review that will synthesise evidence on demographic, psychological/psychiatric, social, cultural-historical, and structural risk factors associated with non-fatal self-harm and suicide attempts among AI/AN adolescents and young adults in the United States. It will also examine methods of suicide attempts and indicators of clinical severity or lethality.

**Methods and analysis:**

This systematic review protocol will follow PRISMA-P 2015 guidelines and the completed review will be reported according to PRISMA 2020. Electronic databases MEDLINE via PubMed, Embase, PsycINFO, CINAHL and Web of Science Core Collection will be searched from inception. Observational and epidemiological studies reporting non-fatal self-harm or suicide attempts among AI/AN youth will be eligible. Two reviewers (AS&YZ) will independently screen studies, extract data, and assess methodological quality using Joanna Briggs Institute critical appraisal tools. A narrative synthesis will be undertaken, and random-effects meta-analysis will be conducted where appropriate. Certainty of evidence will be evaluated using the GRADE approach. This systematic review protocol has been prospectively registered with the Open Science Framework (OSF) Registries (Registration ID: rjf2u; https://osf.io/rjf2u).

**Discussion:**

This review will provide the first synthesis integrating multilevel risk factors with method-specific lethality and clinical severity patterns among AI/AN adolescents and young adults aged 10–25 years. The findings are expected to inform culturally responsive prevention strategies, guide Tribal and Indian Health Service planning, and strengthen equity-focused public health efforts aimed at reducing disparities in suicide attempts among AI/AN adolescents and young adults.

## Background

Suicide is a major global public health concern, with more than 720,000 deaths worldwide each year [1]. It is among the leading causes of death among adolescents and young adults globally, contributing substantially to premature mortality during critical developmental periods [1]. In the United States, suicide remains a major public health concern, accounting for 48,824 deaths in 2024 [2]. The burden of non-fatal suicidality is substantially greater; in 2024, an estimated 14.3 million U.S. individuals seriously considered suicide, 4.6 million made a suicide plan, and 2.2 million attempted suicide [2].. These figures highlight the substantial burden of non-fatal suicidal behavior and underscore the importance of examining suicide attempts as a distinct and clinically meaningful outcome.

Although suicide affects all demographic groups, marked disparities exist across racial and ethnic populations. Among these, non-Hispanic American Indian and Alaska Native (AI/AN) populations consistently experience the highest suicide rates in the United States [2,3]. Between 2015 and 2020, suicide rates among AI/AN individuals increased by nearly 20%, compared with less than a 1% increase in the overall U.S. population during the same period [4]. Suicide is the second leading cause of death among AI/AN youth aged 10–24 years, with age-adjusted suicide rates among AI/AN individuals aged 15–24 years substantially exceeding national averages [5].

These disparities are situated within broader structural, historical, and sociocultural contexts. AI/AN communities have experienced the enduring effects of colonization, forced relocation, land dispossession, cultural suppression, and intergenerational trauma [6]. Historical trauma, systemic marginalization, rural isolation, poverty, under-resourced healthcare systems, and exposure to violence contribute to elevated vulnerability to mental health challenges, including suicidal behavior [7–9]. Emerging evidence also suggests that environmental exposures, including higher temperatures, extreme weather events, and air pollution, may contribute to suicidality, although existing evidence predominantly concerns suicide mortality rather than non-fatal suicidal outcomes [10]. Understanding suicide attempts within this context requires attention not only to individual-level psychological factors but also to social, cultural, structural, and historical determinants, including the legacy of colonization.

Focusing specifically on suicide attempts and self-harm is critical for prevention. Suicide attempts are among the most robust predictors of subsequent suicide death, with evidence suggesting that approximately 10% of individuals who survive a suicide attempt later die by suicide [11]. Importantly, suicidal ideation and suicide attempts are distinct phenomena, and the transition from suicidal thoughts to suicidal behavior involves factors beyond those associated with the emergence of suicidal ideation [12].

In addition to identifying risk factors, understanding the methods used in suicide attempts is essential [13]. Method choice is a critical determinant of case fatality and medical severity, with substantial variation in lethality across methods [14]. For instance, hanging had the highest case fatality rate followed by jumping from heights and drowning [14]. Patterns of method use are shaped by contextual factors, including access to means, geographic location, and sociocultural norms. Examining method distribution and indicators of clinical severity, such as emergency department presentation, hospitalization, intensive care admission, and case fatality, can inform culturally responsive and context-specific prevention strategies, including means safety initiatives.

To date, several systematic reviews have examined aspects of suicidality among AI/AN populations [15–19]. However, these reviews have addressed related but distinct domains of inquiry. Burnette and Figley (2016) and Wiglesworth et al. (2022) primarily examined risk and protective factors within broader frameworks of mental health and wellness, rather than focusing specifically on non-fatal self-harm or suicide attempts. Pham et al. (2022) synthesised suicide prevention interventions and theoretical rationales, while Lehti et al. (2009) focused on Arctic Indigenous populations, including non-U.S. groups, limiting direct applicability to AI/AN populations in the United States. Although Fetter et al. (2023) conducted a comprehensive review of risk factors for suicidal behaviors across the lifespan, the review did not disaggregate findings for adolescents and young adults nor examine method choice or indicators of attempt severity.

To our knowledge, no prior systematic review has simultaneously (1) focused specifically on AI/AN adolescents and young adults aged 10–25 years, (2) synthesised multilevel risk factors within a structured framework, and (3) examined method distribution alongside indicators of clinical severity and lethality. This represents a critical gap in the evidence base for AI/AN youth suicide prevention.

The age range of 10–25 years spans early adolescence through emerging adulthood—a developmental period characterized by neurodevelopmental changes, heightened impulsivity, evolving identity formation, and increased exposure to social and environmental stressors [20–22]. Understanding how demographic, psychological, social, cultural, and structural factors interact to influence suicide attempts during this developmental stage is essential for informing timely and developmentally appropriate interventions.

In this review, non-suicidal self-injury (NSSI) and suicide attempts are treated as distinct outcomes based on suicidal intent. NSSI refers to deliberate self-injurious behavior without suicidal intent, whereas a suicide attempt involves at least some intent to die. Because the term ‘self-harm’ is used variably in the literature to encompass behaviors with or without suicidal intent, it is retained when describing studies in which intent is unspecified or cannot be determined.This review will address the following questions among AI/AN adolescents and young adults (10–25 years) in the United States:

1. What demographic, psychological/psychiatric, social, cultural/historical, and structural risk factors are associated with NSSI and suicide attempts?
2. What methods are used in NSSI and suicide attempts?
3. What indicators of clinical severity or lethality (e.g., ED presentation, hospitalization, ICU admission, near-fatal attempts) are reported and how do they vary across methods?

By integrating epidemiological, developmental, and structural perspectives, this review seeks to inform culturally sensitive, and context specific suicide prevention strategies for AI/AN youth.

## Methods

### Protocol design and reporting framework

This protocol describes a systematic review examining risk factors and methods of NSSI and suicide attempts among American Indian and Alaska Native (AI/AN) adolescents and young adults. The protocol has been developed in accordance with the Preferred Reporting Items for Systematic Review and Meta-Analysis Protocols (PRISMA-P) 2015 guidelines (See S1 File). The completed review will be reported following the PRISMA 2020 statement. This systematic review protocol has been prospectively registered with the Open Science Framework (OSF) Registries (Registration ID: rjf2u; https://osf.io/rjf2u)

Any amendments to this protocol will be documented with the date, description of the change, and rationale, and updated in the OSF record.

### Eligibility criteria

Eligibility criteria are structured using a Population–Exposure–Outcome–Study design (PEOS) framework.

### Population

Studies will be eligible if they include American Indian and/or Alaska Native (AI/AN) adolescents and young adults aged 10–25 years residing in the United States. Studies with broader age ranges will be included if results for the target age group are reported separately, or if the mean age of the sample falls within the specified range. Studies involving Indigenous populations outside the United States, or aggregated Indigenous samples without disaggregated AI/AN data, will be excluded. For mixed-race samples, data will be included only if AI/AN participants are reported as a distinct subgroup. AI/AN identification must be based on self-report, caregiver report, tribal enrollment, or administrative records, as defined by the original study.

### Exposure (risk factors)

Eligible studies may examine at least one risk factor associated with NSSI or suicide attempts. Risk and protective factors may include demographic characteristics (e.g., age, sex, gender identity), psychological or psychiatric factors (e.g., depression, anxiety, substance use, trauma exposure), social and interpersonal determinants (e.g., family conflict, peer relationships, social isolation), cultural and historical influences (e.g., cultural identity, historical trauma, cultural continuity), and structural, contextual, or environmental factors (e.g., poverty, housing instability, discrimination, access to healthcare, and environmental or climate-related exposures)

### Outcomes

In this review, NSSI is defined as deliberate self-inflicted injury without suicidal intent, whereas suicide attempts involve self-injurious behavior with at least some intent to die, as defined by the original study authors. The term *self-harm* is used inconsistently in the literature and may refer to non-suicidal self-injury (NSSI), suicide attempts, or both [23,24]. When studies use the term “self-harm” without clearly specifying intent, the authors’ operational definitions will be examined and coded accordingly. Where intent is unclear or outcomes are combined, this will be documented and considered in the interpretation of findings.

The primary outcomes are NSSI and suicide attempts among AI/AN adolescents and young adults. These outcomes will be extracted and synthesized separately. Studies reporting self-harm with unclear intent or combined NSSI and suicide-attempt outcomes without separable data will be synthesized separately in the narrative analysis.

Secondary outcomes include methods of NSSI and suicide attempts and indicators of severity or lethality. These may include method-based potential lethality (e.g., firearms, hanging, poisoning), clinical severity (e.g., emergency department presentation, hospitalization, intensive care admission), and case fatality or near-fatal outcomes. Severity will be operationalised based on reported clinical indicators and author-defined lethality classifications. Healthcare-utilisation indicators such as emergency department presentation, hospitalization, and ICU admission will not be interpreted as direct measures of lethality in isolation, as they may also reflect geographic accessibility, availability of higher-level care, institutional practices, and healthcare resources. Where reported, these contextual factors will be considered when interpreting clinical severity. Studies focusing exclusively on suicidal ideation or suicide deaths without reporting suicide attempts or NSSI will be excluded.

### Study design and report characteristics

Eligible designs include observational and epidemiological studies (cross-sectional, cohort, case–control), surveillance-based studies, and secondary analyses of national or regional datasets. Mixed-methods studies will be included if quantitative data relevant to the review outcomes can be extracted. Qualitative-only studies, case reports, case series, editorials, commentaries, dissertations, conference abstracts, and intervention trials without baseline observational data will be excluded.

Only peer-reviewed journal articles will be eligible. Restricting inclusion to peer-reviewed articles is based on the need for sufficient methodological detail for risk-of-bias assessment; however, this may exclude relevant evidence from tribal reports and other grey literature, which will be acknowledged as a limitation.

No language restrictions will be applied during the database search in order to minimize the risk of language bias. Studies published in languages other than English will be assessed during the screening stage. Where feasible, non-English articles will be translated using available translation tools or professional translation services. However, given that research involving American Indian and Alaska Native populations in the United States is predominantly published in English, it is anticipated that most eligible studies will be English-language publications.

### Information sources

The following electronic databases will be searched: MEDLINE (via PubMed), Embase, PsycINFO, CINAHL and Web of Science Core Collection. In addition, the reference lists of included studies and relevant systematic reviews will be manually screened to identify additional eligible studies.

Grey literature sources will not be systematically searched, as the review aims to synthesize peer-reviewed research evidence. This decision is based on feasibility constraints and the need for sufficient methodological detail for risk-of-bias assessment; however, this may exclude relevant tribal or community-based reports and will be acknowledged as a limitation.

### Study status and timeline

At the time of manuscript submission, the systematic review has not yet begun the screening or data extraction stages and no results have been generated. The electronic database search is planned to commence on April 1, 2026, with the final search expected to be completed no later than August 1, 2026. Title and abstract screening will follow the search process, with full-text screening, data extraction, and risk-of-bias assessment conducted subsequently. Data synthesis and preparation of results are expected to be completed by August 2026.

### Search strategy

A comprehensive search strategy for each electronic databases will be developed in consultation with an experienced health sciences librarian (OF). The strategy will combine controlled vocabulary (e.g., MeSH and Emtree terms) and free-text keywords relating to: American Indian and Alaska Native populations; Adolescents and young adults and; NSS, Self-harm and suicide attempts

The search strategy developed for PubMed is provided in S2 File and will be adapted appropriately for the remaining databases.

Each database will be searched from inception to the date of the final search, with no publication-date restrictions applied.. Search strategies for all databases will be reported in full in the final publication to ensure reproducibility.

### Data management

All search results will be exported in RIS format and uploaded into Rayyan for deduplication and screening. Rayyan’s automated duplicate detection function will be used, followed by manual verification to ensure accuracy. The de-duplicated dataset will then be used for title/abstract and full-text screening. Audit trails of screening decisions will be maintained within Rayyan

### Selection process

Titles and abstracts will be screened independently by two reviewers (AS & YZ) against the eligibility criteria. Full texts of potentially eligible studies will be retrieved and assessed independently by the same reviewers. Disagreements will be resolved through discussion, with consultation of a third reviewer if consensus cannot be reached. The study selection process will be documented using a PRISMA flow diagram.

### Data collection process

A standardised data extraction form will be developed and pilot-tested on a subset of included studies. Data extraction will be conducted independently and in duplicate by two reviewers (AS & YZ). Where necessary, study authors may be contacted for clarification or missing information. Any discrepancies in extracted data will be resolved by consensus.

### Data items

The following variables will be extracted:

- Study characteristics (author, year, study design, setting)
- Sample characteristics (age range, mean age, sex distribution, tribal affiliation where reported)
- Definitions and measurement of, NSSI and suicide attempts
- Risk factors examined and corresponding effect estimates (e.g., odds ratios, relative risks)
- Methods of NSSI and suicide attempts
- Indicators of severity or lethality (clinical outcomes, healthcare utilisation, case fatality)

Where data are missing or reported inconsistently, assumptions and simplifications will be explicitly documented. Where multiple effect estimates are reported for the same association, adjusted estimates will be prioritised over unadjusted estimates, and the most fully adjusted model will generally be extracted. If multiple models are reported, extraction decisions will be pre-specified in the data extraction form and applied consistently.

### Outcomes and prioritisation

The primary outcomes are risk factors associated with NSSI and suicide attempts. The secondary outcomes are methods of NSSI and suicide attempts and indicators of severity or lethality. Outcomes are prioritised based on their relevance for informing culturally responsive suicide prevention strategies for AI/AN adolescents and young adults.

### Risk of bias assessment

Methodological quality will be assessed independently by two reviewers (AS&YZ) using the appropriate Joanna Briggs Institute (JBI) critical appraisal checklists for cohort, case–control, and cross-sectional studies, as applicable to each included study design. Disagreements will be resolved through discussion or consultation with a third reviewer. Risk-of-bias assessments will inform the interpretation of findings. Studies at high risk of bias will not be excluded but will be subjected to sensitivity analysis and their influence on conclusions will be explicitly discussed.

### Data synthesize

A narrative synthesize will be undertaken due to anticipated heterogeneity in study designs, outcome definitions, and measurement of risk factors. Findings will be grouped thematically according to categories of risk factors and methods of NSSI and suicide attempts.

Quantitative synthesize (meta-analysis) will be considered if a sufficient number of studies report comparable outcomes using similar definitions and effect measures. If conducted, pooled estimates (e.g., odds ratios) will be calculated using random-effects models, and heterogeneity will be assessed using the I² statistic. Thresholds for heterogeneity will be interpreted as low (<25%), moderate (25–75%), or high (>75%), and sources of heterogeneity will be explored narratively or through subgroup analyses where feasible. Subgroup analyses will be conducted where data permit, comparing adolescents (10–19 years) and young adults (20–25 years).

When individual studies report multiple effect estimates derived from overlapping samples (e.g., multiple risk factors, stratified analyses, or partially overlapping subgroups), a single estimate will be selected based on pre-specified criteria (e.g., the most fully adjusted model or the most developmentally relevant subgroup) to avoid double-counting participants.

Where multiple publications analyse the same underlying dataset (e.g., Youth Risk Behavior Surveillance System [YRBSS]) and include overlapping samples, publications examining different risk factors, outcomes, time periods, or population subgroups may be retained if they provide distinct relevant data. However, overlapping participants will not contribute more than once to the same pooled analysis. Where publications report the same exposure–outcome association using overlapping samples, the study with the largest relevant sample, most complete data, and most fully adjusted analysis will be prioritised. Any remaining uncertainty regarding sample overlap will be documented and considered in the interpretation of findings..

### Meta-bias assessment

If ten or more studies are included in a quantitative synthesize, publication bias will be assessed using funnel plots and statistical tests for asymmetry. Where meta-analysis is not feasible, potential reporting biases will be considered narratively.

### Confidence in cumulative evidence

The overall certainty of evidence for each primary outcome will be assessed using the GRADE (Grading of Recommendations Assessment, Development and Evaluation) approach. Evidence will be rated as high, moderate, low, or very low based on risk of bias, consistency, directness, precision, and publication bias.

As the included evidence is expected to be predominantly observational, certainty ratings for most associations will initially be considered as low and may be rated up or down based on standard GRADE criteria (e.g., large effect sizes, dose–response gradients, serious risk of bias, inconsistency, or imprecision).

## Discussion

This systematic review will synthesize evidence on risk factors, methods, and clinical severity of suicide attempts among American Indian and Alaska Native (AI/AN) adolescents and young adults aged 10–25 years. To our knowledge, this will be the first systematic review to integrate multilevel risk factors with methods and indicators of clinical severity or lethality within this age group among AI/AN populations in the United States. By considering individual-level factors alongside broader social, cultural, structural, and health-system contexts, the review may provide a more comprehensive understanding of NSSI and suicide attempts among AI/AN adolescents and young adults.

At the global level, the findings align with the World Health Organization’s suicide prevention framework, which emphasises early identification of risk, restriction of access to lethal means, and targeted strategies for high-risk populations [25]. Nationally, the review supports priorities outlined in the 2024 U.S. National Strategy for Suicide Prevention, including equity-focused prevention, strengthening crisis care systems, and reducing disparities among disproportionately affected groups [26].

Evidence regarding attempt methods and clinical severity may inform implementation of the 988 Suicide & Crisis Lifeline, particularly efforts to enhance culturally responsive crisis services for Tribal communities [27]. The review also has relevance for Indian Health Service (IHS) Zero Suicide initiatives and the Garrett Lee Smith Youth Suicide Prevention Program, which emphasise youth-focused, community-based, and data-driven prevention strategies [28].

By identifying modifiable risk factors, developmental patterns, and method-specific lethality profiles, this review may inform federal and Tribal planning, guide resource allocation within IHS and Tribal health systems, and strengthen coordinated prevention strategies. Importantly, findings will be interpreted within the broader context of historical oppression, structural inequities, and chronic under-resourcing of Tribal health systems. Synthesizing method-specific lethality patterns may also support culturally responsive lethal-means safety counselling, suicide risk assessment, and post-attempt clinical pathways in both Tribal and urban Indian health settings. To strengthen the cultural and contextual interpretation of the findings, the synthesized results will be shared with an AI/AN community partner with whom the research team has an established partnership, and feedback will be sought on the interpretation and contextualization of the findings prior to final dissemination.

### Strengths and limitations

This systematic review protocol has several methodological strengths, including adherence to PRISMA-P guidelines, duplicate screening and data extraction, use of established risk-of-bias tools, and a structured approach to managing definitional heterogeneity and overlapping datasets. The defined age range of 10–25 years and explicit distinction between NSSI and suicide attempts enhance conceptual clarity. However, the review is limited to peer-reviewed studies, which may exclude relevant Tribal reports, community surveillance data, culturally specific evaluations, and other community-generated knowledge. This exclusion may limit the cultural and contextual comprehensiveness of the evidence synthesized and should be considered when interpreting the findings across diverse AI/AN communities. Variability in outcome definitions, measurement approaches, and reporting of effect estimates may limit comparability across studies and reduce the feasibility of quantitative synthesis. Additionally, reliance on observational data may constrain causal interpretation of identified associations.

## Supporting information

S1 File Appendix A. (DOCX)

S2 File Appendix B. (DOCX)
