# Supplementary material for: Risk factors and methods of NSSI and suicide attempts among American Indian and Alaska native adolescents and young adults: A systematic review protocol
Source: PLoS One. 2026 Sep 15;21(9):e0358536. doi: 10.1371/journal.pone.0358536 (PMC13577370; doi:10.1371/journal.pone.0358536)
Supplement: Supplementary file 2 [file pone.0358536.s002.docx]

**Appendix B PubMed Search Strategy**

The following search strategy was developed for MEDLINE via PubMed and will be adapted for other databases as appropriate.

1. ("Indians, North American"[MeSH]

OR "Alaska Natives"[MeSH]

OR "American Indian*"[Title/Abstract]

OR "Native American*"[Title/Abstract]

OR "Alaska Native*"[Title/Abstract]

OR "American Indian and Alaska Native*"[Title/Abstract]

OR "American Indians and Alaska Natives"[Title/Abstract]

OR "Indigenous American*"[Title/Abstract]

OR "AI/AN"[Title/Abstract])

2. ("Adolescent"[MeSH]

OR "Young Adult"[MeSH]

OR "Child"[MeSH]

OR adolescent*[Title/Abstract]

OR youth*[Title/Abstract]

OR teen*[Title/Abstract]

OR "young adult*"[Title/Abstract]

OR child*[Title/Abstract]

OR "young people"[Title/Abstract]

OR "emerging adult*"[Title/Abstract])

3. ("Suicide, Attempted"[MeSH]

OR "Self-Injurious Behavior"[MeSH]

OR "suicide attempt*"[Title/Abstract]

OR "self-harm*"[Title/Abstract]

OR "self injur*"[Title/Abstract]

OR "self-injur*"[Title/Abstract]

OR NSSI[Title/Abstract]

OR "non-suicidal self-injur*"[Title/Abstract]

OR "nonsuicidal self-injur*"[Title/Abstract])

4. 1 AND 2 AND 3
